# It’s never too late - balance and endurance training improves functional performance, quality of life, and alleviates neuropathic symptoms in cancer survivors suffering from chemotherapy-induced peripheral neuropathy: results of a randomized controlled trial

**DOI:** 10.1186/s12885-019-5522-7

**Published:** 2019-05-02

**Authors:** S. Kneis, A. Wehrle, J. Müller, C. Maurer, G. Ihorst, A. Gollhofer, H. Bertz

**Affiliations:** 1 Department of Medicine I, Faculty of Medicine and Medical Center, University of Freiburg, Hugstetterstr. 55, 79106 Freiburg, Germany; 2 Institute for Exercise- and Occupational Medicine, Faculty of Medicine and Medical Center, University of Freiburg, Freiburg, Germany; 3 Department of Sport and Sport Science, University of Freiburg, Freiburg, Germany; 4 Department of Neurology and Neuroscience, Faculty of Medicine and Medical Center, University of Freiburg, Freiburg, Germany; 5 Clinical Trials Unit, Faculty of Medicine and Medical Center, University of Freiburg, Freiburg, Germany; 6 Present address: Department of Medical Oncology, National Center for Tumor Diseases (NCT), Heidelberg, Germany

**Keywords:** Peripheral nervous system diseases, Somatosensory disorders, exercise therapy, Postural balance, Neuromuscular adaptation, Quality of life

## Abstract

**Background:**

Chemotherapy-induced peripheral neuropathy (CIPN) can affect functional performance and quality of life considerably. Since balance training has proven to enhance physical function, it might be a promising strategy to manage CIPN-induced functional impairments.

**Methods:**

Fifty cancer survivors with persisting CIPN after finishing their treatment were randomly allocated to an intervention (IG) or active control group (CG). The IG did endurance plus balance training, the CG only endurance training (twice weekly over 12 weeks). Pre- and post-assessments included functional performance, cardiorespiratory fitness, vibration sense, and self-reported CIPN symptoms (EORTC QLQ-CIPN20).

**Results:**

Intention-to-treat analyses (*n* = 41) did not reveal a significant group difference (CG minus IG) for sway path in semi-tandem stance after intervention (primary endpoint), adjusted for baseline. However, our per-protocol analysis of 37 patients with training compliance ≥70% revealed: the IG reduced their sway path during semi-tandem stance (− 76 mm, 95% CI -141 – -17; CG: -6 mm, 95% CI -52 – 50), improved the duration standing on one leg on instable surface (11 s, 95% CI 8–17; CG: 0 s, 95%CI 0–5) and reported decreased motor symptoms (−8points, 95% CI -18 – 0; CG: -2points 95% CI -6 – 2). Both groups reported reduced overall- (IG: -10points, 95% CI -17 – -4; CG: -6points, 95% CI -11 – -1) and sensory symptoms (IG: -7points, 95% CI -15 – 0; CG: -7points, 95% CI -15 – 0), while only the CG exhibited objectively better vibration sense (knuckle: 0.8points, 95% CI 0.3–1.3; IG: 0.0points, 95% CI -1.1 – 0.9; patella: 1.0points, 95% CI 0.4–1.6: IG: -0.8points, 95% CI -0.2 – 0.0). Furthermore, maximum power output during cardiopulmonary exercise test increased in both groups (IG and CG: 0.1 W/kg, 95% CI 0.0–0.2), but only the CG improved their jump height (2 cm, 95% CI 0.5–3.5; IG: 1 cm, 95% CI -0.4 – 3.2).

**Conclusion:**

We suppose that endurance training induced a reduction in sensory symptoms in both groups, while balance training additionally improved patients’ functional status. This additional functional effect might reflect the IG’s superiority in the CIPN20 motor score. Both exercises provide a clear and relevant benefit for patients with CIPN.

**Trial registration:**

German Clinical Trials Register (DRKS) number: DRKS00005419, prospectively registered on November 19, 2013.

## Background

Peripheral neuropathy symptoms often persist after chemotherapy treatment has finished, and they can significantly impair patients’ quality of life, even in the long term [1]. The prevalence of chemotherapy-induced peripheral neuropathy (CIPN) can total 68% during the first month after the end of chemotherapy [2], and its consequences are known to trigger excessive healthcare costs and resource use [3].

Affected patients suffer from symptoms like pain and paraesthesia, loss of sensation and proprioception in the lower extremities resulting in muscle weakness, balance problems, and gait instability may lead to a higher risk of falling [4]. Such functional impairments can substantially limit mobility [5] and even predict hospitalization or mortality [6]. Based on the ASCO guidelines, only duloxetine can currently be recommended for pain reduction in CIPN [7]. The efficacy of further pharmacological and non-pharmacological approaches is not evidence-based [7]. Therefore, we pursue further effective treatment options to ensure patients’ social participation by preserving their mobility and reducing health risks that entail a prolonged need for therapy. There is cross-etiological evidence that exercising can reduce neuropathic symptoms [8]: patients with diabetic neuropathy benefit from exercising like endurance [9, 10], balance [11, 12] and multimodal training [13, 14]. Endurance training induces metabolic changes, and balance training [8] leads to neuronal adaptations and improved muscular output resulting in a better postural control [15, 16]. Concerning CIPN, exercising is generally recommended [4] but has been less evaluated [17]. Our intervention study on lymphoma patients provided initial indications about exercising and CIPN, where we speculated that especially balance exercises would reduce CIPN sensory symptoms and improve physical functioning [18]. In our subsequent pilot study, exclusively CIPN patients underwent the aforementioned intervention and benefited from exercising by approximating the posture behavior of matched healthy control subjects (data unpublished). We thus implemented the present trial to evaluate exercise effects on CIPN symptoms and functional performance. Our primary objective was to improve CIPN patients’ balance performance, hypothesizing that balance exercises would lead to a reduction in postural sway after a twelve-week intervention.

## Methods

### Study design and patients

Fifty cancer survivors were randomly allocated consecutively between December 2013 and November 2014 to an intervention group (IG) or active control group (CG). Randomization in blocks of 10 was based on a computer-assisted pseudo-random number generator (Research Randomizer, Version 4.0). Allocation was implemented by sequentially numbered, sealed, opaque envelopes. After obtaining patient’s consent, baseline measurement was performed and the next consecutively numbered envelope was opened afterwards.

Inclusion criteria were: reporting CIPN symptoms, completion of anti-tumor treatment, ≥18 years, a maximum 90 min’ travel time to the Medical Center – University of Freiburg, Germany, and written informed consent. Exclusion criteria were: neuropathies of different origin, severe cardiovascular diseases, instable bone metastases, and pregnancy. Pre- and post-assessments were made before (T0) and after (T1) intervention and took place at the Institute for Exercise- and Occupational Medicine, Medical Center – University of Freiburg, Germany.

Lower-extremity CIPN was clinically confirmed by assessing reflexes and vibration sense and by discrimination tests for joint position sense, temperature, and pain sensation (Table 1).

**Table 1:** Patients’ characteristic

	Intention-to-treat		Per-protocol			
	All *N* = 41	IG vs. CG *P*	IG *N* = 18	CG *N* = 19	All *N* = 37	IG vs. CG *P*
Age						
median (range)	62 (44 – 82)	**0.025**	70 (44 – 82)	60 (46 – 75)	63 (44 – 82)	**0.026**
Sex						
m:f *N* (%)	11 (27): 30 (73)		4 (22): 14 (78)	7 (37): 12 (63)	11 (30): 26 (70)	
BMI						
median (range)	25 (19 – 42)	0.375	25 (19 – 42)	24 (21 – 31)	24 (19 – 42)	0.425
Diagnosis *N* (%)						
Breast cancer	14 (34)		8 (44)	4 (21)	12 (32)	
Colorectal cancer	14 (34)		3 (17)	10 (53)	13 (35)	
Gynecological cancer other than breast	4 (10)		2 (11)	1 (5)	3 (8)	
Upper gastrointestinal cancer	2 (5)		1 (6)	1 (5)	2 (5)	
Non-small cell lung cancer	1 (2)		0 (0)	1 (5)	1 (3)	
Non-Hodgkin’s lymphoma	5 (12)		4 (22)	1 (5)	5 (14)	
Multiple myeloma	1 (2)		0 (0)	1 (5)	1 (3)	
Therapies *N* (%)						
Surgery	38 (93)		16 (89)	18 (95)	34 (92)	
Radiation	15 (37)		8 (44)	5 (26)	13 (35)	
Hematopoietic cell transplantation	2 (5)		1 (6)	1 (5)	2 (5)	
Chemotherapy	41 (100)		18 (100)	19 (100)	37 (100)	
*N* cycles, median (range)	6 (1 – 18)	0.681	6 (2 – 6)	6 (2 – 16)	6 (2 – 16)	0.775
*N* neurotoxic agents, median (range)	2 (1 – 4)	0.115	2 (1 – 4)	1 (1 – 4)	2 (1 – 4)	0.061
Therapy-free weeks, median (range)	11 (1 – 167)	0.754	10 (1 – 167)	18 (3 – 98)	13 (1 – 167)	0.775
CIPN symptoms *N* (%)						
Reduced vibration sense*	28 (70)		13 (77)	13 (68)	26 (72)	
Reduced joint position sense^#^	11 (28)		5 (29)	6 (32)	11 (31)	
Reduced temperature sensation^†^	23 (58)		12 (71)	10 (53)	20 (56)	
Reduced pain sensation^†^	4 (10)		2 (12)	2 (11)	4 (11)	
Loss of reflexes ASR / PSR	28 (68) / 8 (20)		12 (67) / 3 (17)	13 (68) / 3 (16)	25 (68) / 6 (16)	
Compliance %, median (range)	92 (25 – 100)	0.175	92 (71 – 100)	100 (71 – 100)	96 (71 – 100)	0.118

*measured on the first metacarpophalangeal joint, value < 5 (scale 0–8); ^#^measured on second toe, ≥ 3 failures out of 10 trials in random order; ^†^measured on arch, ≥ 3 failures out of 10 trials in random order; bold type indicates significance *P*<0.05

Abbreviations: *ASR* Achilles tendon reflex, *PSR* patellar tendon reflex, *IG* intervention group, *CG* control group, *BMI* body mass index, *CIPM* chemotherapy-induced peripheral neuropathy

This study was approved by the Ethics Committee of the University of Freiburg, conducted according to the Declaration of Helsinki and registered in the German Clinical Trials Register (DRKS00005419).

### Interventions

The one-on-one training sessions took place twice per week over 12 weeks in the division of Sports Oncology in the Clinic of Internal Medicine I. Both groups underwent endurance training up to 30 min of moderate intensity below the individual anaerobic threshold (IAT) on a stationary bicycle. The IG also did 30 min’ balance training. Balance exercise sessions included three to eight exercises with three repetitions each à 20 – 30s involving progressively increasing exercise difficulty by reducing the support surface and visual input, adding motor/cognitive tasks, and instability induction [19].

For both groups, we additionally monitored exercise intensity by the perceived exertion rating scale [20, 21].

Furthermore, we controlled each patient’s blood pressure and heart rate during each training session to avoid overload and documented vital parameters, training progress and reasons for missed sessions.

### Outcome measures

#### Functional performance

All the measurements were performed on a force plate (Leonardo Mechanograph® GRFP, Novotec Medical GmbH, Pforzheim, Germany), which determined dynamic ground reaction forces in its local and temporal progress. For balance assessments, we recorded the center of force sway path (mm) during three different stance conditions: semi-tandem stance with eyes open (ST_EO_) (primary endpoint) and eyes closed (ST_EC_), and monopedal stance (MS_EO_) over a period of 30s with a sample rate of 800 Hz. While measuring, patients were asked to stand upright and comfortably and direct their gaze onto a marked spot located at eye level on the wall. The best trial out of three was used for analysis. A reduction of sway path after exercising is associated with an improved postural control.

Additionally, we recorded the duration (max. 30s) patients could stand on one leg on a stable (MS_EO_) and unstable (MS_EOunstable_) surface, respectively.

To evaluate the lower body’s muscle power, patients performed a maximum counter-movement jump to measure maximum power output during take-off per kilogram body weight (P_max_jump_; W/kg) and jumping height (cm). Patients were instructed to jump as high as possible. The best trial of two trials was used for analysis.

Data were analyzed using Leonardo Mechanography Research-Software (Novotec Medical GmbH, Pforzheim, Germany).

#### CIPN symptoms and quality of life

Vibration sense was determined on the first metacarpophalangeal joint, knuckle and patella via Rydel-Seiffer tuning fork with a graduating scale from 0 (no sensitivity) to 8 (highest sensitivity); due to reliability, tests were repeated twice, the respective mean value was used for analysis. For patients’ characteristic, reduced vibration sense was defined as < 5 [22].

We used the EORTC QLQ-C30-questionnaire (European Organization for Research and Treatment of Cancer Quality of Life) to assess global quality of life (QoL). A higher score (max 100%) represents a higher quality of life [23]. The module EORTC QLQ-CIPN20 and neurotoxicity subscale (NtxS) of FACT&GOG (Functional Assessment of Cancer Therapy/Gynaecology Oncology Group) were used to estimate CIPN severity. For CIPN20, we calculated a sum score and five sub-scores (sensory, motor, autonomic, upper and lower extremity). Each sub-score ranges from 0 to 100, where higher scores represent more severe symptoms or impairment.

#### Cardiorespiratory fitness

We determined cardiorespiratory fitness by peak oxygen consumption (V̇O_2peak_; mL·min^− 1^**·**kg^− 1^), maximum power output (P_max_CPET_; W/kg) and performance at the IAT (W/kg) measured during the maximum cardiopulmonary exercise test (CPET). CPET [24] including electrocardiogram and blood pressure measurement took place on an electronically-braked cycle ergometer (Ergoline 900, Bitz, Germany) in recumbent position, starting at 20 watt and increasing stepwise by 10 watt every minute until exhaustion [21]. Gas exchange and ventilation was continuously recorded by a breath-by-breath gas analysis system (Oxycon Delta, Jaeger, Hochberg, Germany). IAT was determined by analyzing the lactate concentration per step (Ergonizer, Freiburg, Germany).

### Sample size and statistics

**Fig. 1:**
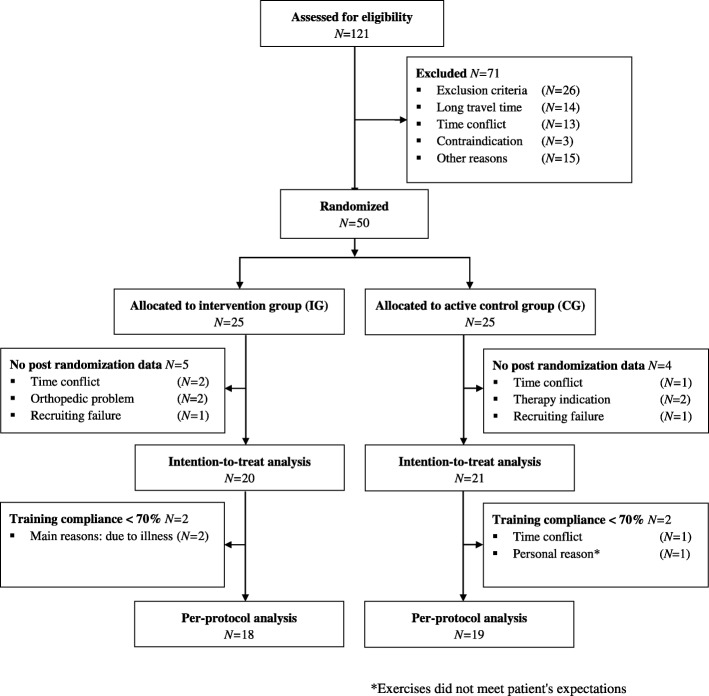
Flow diagram for participants included in study

Sample size calculation is based on the primary endpoint sway path at T1 and aims to detect a mean difference of 30% (SD ± 32%) between groups according to pilot study results. For sample size purposes, sway path is calculated as % of baseline measurement. With these prerequisites, 20 patients per group are required to provide 80% power to obtain a significant study result, using the 2-sided *t*-test with α = 0.05. Considering a maximum dropout rate of 20%, total sample size was set to *N* = 50. As specified in the clinical trial protocol, our primary analysis was conducted via regression model for variable ST_EO_ at T1 as dependent variable, treatment allocation and baseline ST_EO_ as covariates. Patients on whom we had no post-randomization data were excluded from the intention-to-treat analysis (Fig. 1 Flowchart). A sensitivity analysis of the primary endpoint included the therapy-free time until study inclusion and patient age as additional covariates.

We also conducted a per-protocol analysis that excluded patients with training compliance < 70%, calculated as completed training sessions divided by planned training sessions. All variables were tested non-parametrically as the assumption of normal distribution (Shapiro-Wilk test) was not satisfied. Differences between our two subject subpopulations at T0 and T1 and differences in the groups’ delta (T1-T0) were assessed by Mann-Whitney-U-test. Intragroup differences over time were computed by Wilcoxon signed-rank test. The level of significance was set to *p* < .05. To estimate the treatment effect, the point estimate and 95% confidence interval (CI) of the Hodges-Lehmann’s median differences for paired groups were used. We also calculated the Phi coefficient (r_φ_= $\sqrt{z2/n}$) for effect sizes based on z-statistics of Wilcoxon- and Mann-Whitney-U test, respectively [25]. IBM SPSS software (version 24; SPSS Inc., Chicago, Illinois, USA) was used for all analyses.

## Results

No adverse events were observed during the study period. As post-randomization data were unavailable on seven patients, and two patients were excluded due to recruiting failure, our intention-to-treat analyses (ITT) included 41 patients. The primary analysis linear regression model (ITT) did not reveal a sway-path group difference (CG minus IG) at T1 (estimated as 35 mm; 95%CI -30 – 101; *p* = .279), adjusted for baseline. The sensitivity analysis revealed that the covariates therapy-free time until study inclusion and patients’ age did not lead to a fundamentally different interpretation of our results (see Table 2 for regression analysis results).

**Table 2:** Influence of group on “STEO sway path (mm) at T1” based on the regression analysis

Model including as additional covariates	Estimated group difference for ST_EO_ sway path (mm) at T1	95% CI	*P*
Primary analysis model (ITT) including			
ST_EO_ sway path at T0	34.5	-30.0 – 100.9	0.279
Sensitivity analysis model including additionally			
Patients’ age	30.4*	-38.1 – 98.8	0.088
Therapy-free time until study inclusion	27.9*	-36.2 – 91.9	0.384
Per-protocol analysis model including			
ST_EO_ sway path at T0	32.5*	-41.1 – 106.0	0.376

Regression analysis according to the intention-to-treat- (n = 41) and per-protocol principle (n = 37); independent variable: group; dependent variable: “STEO sway path (mm) at T1”; *adjusted for STEO sway path at T0

Abbreviations: *STEO* semi-tandem stance with eyes open, *CI* confidence interval

As not all patients attained ≥70% compliance, we present a per-protocol-analysis (*n* = 37) to describe the treatment effect in this group (see Table 3 and the following). We noted similar baseline values in the IG and CG, except for semi-tandem stance with eyes open, monopedal stance on instable surface and jumping performance, where the CG performed better in each case (ST_EO_: *P* = .049; MS_EOunstable_: *P* = .011; P_max_jump_: *P* = .019; Jumping height: *P* = .045).

**Table 3:** Results of per-protocol analysis (*N* = 37)

		T0 median (range)	T1 median (range)	median difference* (95% CI)	*P*	r_ϕ_
Balance performance						
ST_EO_ sway path (mm)	IG	628 (477 – 1339)	616 (420 – 1261)	-76 (-141 – -17)	**0.018**	0.56
	CG	572 (326 – 928)	578 (319 – 809)	-6 (-52 – 50)	0.841	0.05
	*P*	**0.049**	0.327	**0.049**		
ST_EC_ sway path (mm)	IG	1290 (735 – 4661)	1486 (804 – 4885)	-32 (-205 – 142)	0.717	0.09
	CG	1466 (649 – 3089)	1342 (607 – 2736)	39 (-115 – 160)	0.809	0.06
	*P*	0.781	0.408	0.659		
MS_EO_ sway path (mm)	IG	1755 (1255 – 2440)	1667 (1058 – 3216)	-204 (-442 – 178)	0.214	0.41
	CG	1510 (844 – 2701)	1303 (711 – 2266)	-204 (-367 – 2)	0.056	0.51
	*P*	0.311	0.059	1.000		
MS_EO_ duration (sec)	IG	30 (1 – 30)	30 (18 – 30)	1 (0 – 7)	0.051	0.49
	CG	30 (11 – 30)	30 (7 – 30)	0 (0 – 2)	0.500	0.15
	*P*	0.285	0.707	0.230		
MS_EOunstable_ duration (sec)	IG	10 (4 – 30)	30 (5 – 30)	11 (8 – 17)	**0.001**	0.80
	CG	30 (4 – 30)	30 (5 – 30)	0 (0 – 5)	0.223	0.30
	*P*	**0.011**	0.845	**0.000**		
Jumping performance						
P_max_jump_ (W/kg)	IG	23 (15 – 42)	23 (15 – 42)	0.3 (-0.9 – 1.6)	0.569	0.14
	CG	28 (16 – 48)	29 (18 – 49)	1.3 (0.2 – 2.2)	**0.044**	0.50
	*P*	**0.019**	0.058	0.539		
Jumping height (cm)	IG	21 (13 – 46)	22 (13 – 42)	1 (-0.4 – 3.2)	0.127	0.38
	CG	29 (10 – 45)	28 (17 – 47)	2 (0.5 – 3.5)	**0.017**	0.59
	*P*	**0.045**	0.068	0.838		
Vibrations sense (scale 0–8)						
First metacarpophalangeal joint	IG	2.5 (0.0 – 7.5)	3.4 (0.0 – 8.0)	0.4 (-0.3 – 1.3)	0.139	0.36
	CG	4.0 (0.0 – 7.8)	4.5 (0.5 – 6.6)	0.4 (-0.1 – 0.9)	0.083	0.40
	*P*	0.071	0.111	0.950		
Knuckle	IG	3.8 (0.0 – 7.3)	4.1 (0.0 – 8.0)	0.0 (-1.1 – 0.9)	0.977	0.01
	CG	4.9 (0.0 – 7.0)	5.4 (0.0 – 7.0)	0.8 (0.3 – 1.3)	**0.017**	0.55
	*P*	0.471	**0.049**	0.175		
Patella	IG	5.0 (0.0 – 8.0)	4.3 (0.0 – 7.3)	-0.8 (-2.0 – 0.0)	**0.041**	0.50
	CG	4.8 (0.0 – 6.5)	5.6 (0.8 – 7.0)	1.0 (0.4 – 1.6)	**0.002**	0.71
	*P*	0.196	**0.005**	**0.000**		
Quality of life (%)						
Global QoL	IG	63 (17 – 100)	79 (33 – 100)	8 (-4 – 17)	0.082	0.39
	CG	67 (17 – 100)	67 (50 – 100)	4 (-4 – 13)	0.307	0.23
	*P*	0.845	0.221	0.461		
Subjective CIPN symptoms						
CIPN20 sum score^#^	IG	28 (6 – 85)	20 (9 – 69)	-10 (-17 – -4)	**0.007**	0.65
	CG	28 (4 – 70)	24 (4 – 63)	-6 (-11 – -1)	**0.027**	0.52
	*P*	0.782	0.499	0.257		
CIPN20 sensory score^#^	IG	41 (4 – 85)	26 (4 – 74)	-7 (-15 – 0)	**0.028**	0.53
	CG	38 (7 – 74)	30 (7 – 70)	-7 (-15 – 0)	**0.018**	0.56
	*P*	0.935	1.000	0.925		
CIPN20 motor score^#^	IG	25 (0 – 79)	8 (0 – 63)	-8 (-18 – 0)	**0.006**	0.67
	CG	17 (0 – 71)	13 (0 – 62)	-2 (-6 – 2)	0.278	0.26
	*P*	0.546	0.916	0.114		
CIPN20 autonomic score^#^	IG	17 (0 – 83)	0 (0 – 50)	-8 (-17 – 0)	**0.006**	0.65
	CG	14 (0 – 83)	17 (0 – 33)	-6 (-17 – 0)	0.151	0.34
	*P*	0.791	0.118	0.313		
CIPN20 upper extremity score^#^	IG	19 (0 – 91)	14 (0 – 81)	-7 (-14 – 2)	0.063	0.45
	CG	19 (0 – 71)	19 (0 – 62)	-3 (-7 – 1)	0.059	0.45
	*P*	0.961	0.641	0.616		
CIPN20 lower extremity score^#^	IG	33 (8 – 96)	25 (0 – 79)	-13 (-19 – -4)	**0.007**	0.66
	CG	29 (8 – 75)	25 (8 – 71)	-8 (-15 – -2)	**0.014**	0.58
	*P*	0.503	0.964	0.552		
NtxS score^†^	IG	29 (11 – 40)	32 (15 – 41)	3 (1 – 6)	**0.015**	0.59
	CG	31 (16 – 39)	31 (19 – 40)	2 (0 – 4)	0.064	0.42
	*P*	0.620	0.940	0.361		
Cardiorespiratory fitness						
V̇O_2peak_ (mL·min^−1^**·**kg^−1^)	IG	21 (16 – 35)	23 (17 – 33)	0.2 (-1.7 – 1.7)	0.650	0.15
	CG	23 (17 – 54)	25 (16 – 54)	1.4 (-0.4 – 3.5)	0.133	0.35
	*P*	0.369	0.242	0.417		
P_max_CPET_ (W/kg)	IG	1.4 (0.9 – 2.4)	1.5 (0.8 – 2.4)	0.1 (0.0 – 0.2)	**0.025**	0.53
	CG	1.6 (0.9 – 3.4)	1.7 (0.9 – 3.5)	0.1 (0.0 – 0.2)	**0.004**	0.66
	*P*	0.408	0.245	0.443		
IAT (W/kg)	IG	1.1 (0.6 – 2.0)	1.1 (0.6 – 1.8)	0.1 (-0.2 – 0.1)	0.122	0.36
	CG	1.1 (0.5 – 2.6)	1.3 (0.8 – 2.6)	0.1 (0.0 – 0.1)	**0.020**	0.54
	*P*	0.374	0.358	0.707		

Notes: * prescribes the treatment effect by point estimation and 95% confidence interval of the Hodges-Lehmann’s median differences for paired groups; # scoring from 0 (no symptoms) – 100 (severe symptoms); † scoring from 0 (severe symptoms) – 44 (no symptoms); bold type indicates significance *P*<0.05

Abbreviations: *T0* pre intervention, *T1* post intervention, *CI* confidence interval, *P p*-value, *rϕ* Phi coefficient, *STEO/ EC* semi-tandem stance with eyes open/closed, *MSEO/EOunstable* Monopedal stance with eyes open on unstable surface, *Pmax_jump/CEPT* maximum power output during jumping/CEPT, *CEPT* cardiopulmonary exercise test, *QoL* quality of life, *CIPN20* module of the EORTC-QLQ (European Organization for Research and Treatment of Cancer Quality of Life) questionnaire, *NtxS* neurotoxicity subscale of FACT/GOG (Functional Assessment of Cancer Therapy/Gynaecology Oncology Group), *V̇O*_2peak_ peak oxygen consumption, *IAT* individual anaerobic threshold, *IG* interventions group, *CG* control group

### Functional performance

IG’s ST_EO_ sway path decreased significantly (− 76 mm, 95%CI -141 – -17; *p* = .018), while the CG’s was unchanged, leading to a significant difference in groups’ delta (*p* = .049). ST_EC_ sway path revealed no inter- or intragroup changes. In the monopedal stance condition (MS_EO_ sway path), both groups improved descriptively without statistical significance, but with moderate effect sizes (r_φ_ = 0.41; r_φ_ = 0.51, respectively). However, only the IG improved their time standing on one leg (MS_EO_: 1 s, 95%CI 0–7; *p* = .051; MS_EOunstable_: 11 s, 95% CI 8–17; *p* = .001), while the CG maintained their performance level, leading to a significant difference in groups’ delta for MS_EOunstable_ (*p* = .000).

CG improved their maximum jump height significantly (2 cm, 95%CI 0.5–3.5; *p* = .039), while the IG’s failed to change. Maximum power (P_max_jump_) was unaltered.

### CIPN symptoms and quality of life

We detected neither inter- nor intragroup differences in vibration sense measured on the first metacarpophalangeal joint (scale 0–8). However, on the knuckle, the CG increased significantly (0.8, 95% CI 0.3–1.3; *p* = .011) leading to a significant group difference at T1 (*p* = .049). Furthermore, the patella’s vibration sense improved significantly in the CG (1.0, 95% CI 0.4–1.6; *p* = .002), while the IG’s decreased significantly (− 0.8, 95% CI -0.2 – 0.0; *p* = .041), leading to a significant difference at T1 (*p* = .005) and in groups’ delta (*p* = .000).

In NtxS, the IG reported significantly alleviated CIPN symptoms (3, 95% CI 1–6; *p* = .015). Except for the upper extremity sub-score, CIPN20 revealed significant weakening in the IG’s CIPN symptoms (sum score: -10, 95% CI -17 – -4; *p* = .007; sensory score: -7, 95% CI -15 – 0; *p* = .028; motor score: -8, 95% CI -18 – 0; *p* = .006; autonomic score: -8, 95% CI -17 – 0; *p* = .006; lower extremity score: -13, 95% CI -19 – -4; *p* = .007), while the CG’s sum, sensory and lower extremity scores also decreased significantly (− 6, 95% CI -11 – -1; *p* = .027; − 7, 95% CI -15 – 0; *p* = .018; − 8, 95% CI -15 – -2; *p* = .014; respectively). Both groups’ global QoL improved slightly but not significantly.

### Cardiorespiratory fitness

The CG significantly improved their performance at IAT after the intervention (0.1 W/kg, 95%CI 0.0–0.1; *p* = .020; no change for IG *p* = .122). Furthermore, both groups strengthened their maximum power output (IG: 0.1 W/kg, 95%CI 0.0–0.2; *p* = .025; CG: 0.1 W/kg, 95%CI 0.0–0.2; *p* = .004). However, we detected no differences in V̇O_2peak_.

## Discussion

The aim of this randomized controlled clinical trial was to assess the effects of endurance and balance training on CIPN symptoms and the physical function of cancer survivors after treatment. Primary intention-to-treat analysis did not reveal a superiority of balance training contrary to our hypothesis. However, subsequent analysis did not entirely support this finding, since the results of per-protocol analysis (≥70% compliance) including secondary endpoints requires a detailed view. For this analysis, however, the number of patients is under the 20 patients per group required according to the power analysis. Our results may have been more convincing with a larger number of patients.

In general, balance training is known to induce neuronal adaptations and improve muscular output leading to an enhanced postural control [15, 16]. It is well known that patients with a proprioceptive deficit such as peripheral neuropathy suffer from postural instability [5], as do patients with CIPN [26–31]. However, only four randomized controlled trials have been published on the effects of balance interventions in CIPN patients [18, 32–34]. Our trial showed that our IG prolonged their standing time on one leg, and reduced their sway path in the semi-tandem stance with eyes open – factors associated with better postural control [28]. Even our CG slightly improved their balance performance in the monopedal stance without having practiced this task. This improvement could be traced back to a general increase in leg-muscle strength induced by endurance training, a factor also reflected by our finding that both groups enhanced their maximum power output during CEPT. However, only CG’s jumping performance increased. Since both groups formally completed the same endurance training, such a change should probably have been observed in both groups. It is conceivable that the CG engaged more intensively in their endurance training, since their training program consisted exclusively of endurance training, which may unconsciously lead to more intense training, while the IG may have considered the 30-min endurance exercise to be a mere warm-up. A further explanatory viewpoint lies in the baseline differences; the CG exhibited greater power capacity already at T0, ie, P_max_jump_ and jump height, than did the IG.

This baseline difference may be attributable to the CG’s younger age, since the rate of force development is known to decline with age [35]. The CG’s younger age may also be responsible for the significant baseline difference in two balance tasks, MS_EOunstable_ and ST_EO_. Their predominant initial functional status may also be because they received a lower amount of neurotoxic agents.

In the eyes-closed condition in the balance tasks, we detected no inter- or intragroup differences, but the sway path increased considerably after closing the eyes. The increase in postural sway when visual information is unavailable is more pronounced in patients with neuropathy than in healthy subjects [5]. These patients may rely more on vestibular signals, which are known to carry a larger amount of noise [36] than on diminished proprioception to stabilize the posture. At this point, we cannot conclusively clarify how severely diminished our patients’ proprioception was, as we did not compare their balance performance to healthy subjects, especially the rise in sway from eyes open to closed. Most of our patients suffered from a reduced vibration sense and reported having more sensory than motor symptoms. Axon degeneration in unmyelinated distal nerve endings seems to be the central pathology of CIPN [37], responsible especially for sensory symptoms [38]. However, we assume that stimulus conduction is not completely dysfunctional: large myelinated nerve fibers carrying proprioceptive information and inducing muscular output might be less affected. Additionally, exercising may have stimulated the use of less damaged pathways. The increase in maximum power output in both groups and their improvements in balance performance might support this hypothesis and indicate that neuromuscular adaptation is possible. However, we observed no improvements in the eyes-closed conditions, which made us conclude that patients did not change their posture strategy towards reducing vestibular in favor of proprioceptive cues. We thus suggest focusing even more strongly on exercises without visual input during training. Being aware that analyzing CIPN20 sub-scores remains controversial [39], our motor-score results may reflect neuromuscular adaptation, as our IG improved considerably. Interestingly, both groups experienced reduced sensory symptoms and greater improvements in their lower extremities, since both exercises obviously targeted the lower body more strongly than the upper. However, objectively, only in the CG did we detect a significantly improved vibration sense from proximal to distal - probably attributable to their lower exposure to neurotoxic agents. Animal models have shown that increased blood flow, and an enhanced overall metabolic rate thanks to endurance training might result in higher levels of neurotrophic factors that may induce nerve regeneration [40, 41] and thus possibly reduce sensory symptoms. Furthermore, the anti-inflammatory effect of exercise might have contributed to weaker sensory symptoms [41].

Endurance training did not just affect CIPN-specific symptoms - it also resulted in improved performance in the CG’s IAT, presumably because of their more intensive endurance training as mentioned above. This increase in endurance capacity was not confirmed in our V̇O_2peak_ findings. Both groups improved their maximum performance during CPET, possibly due to a general strength increase. This strength increase is also apparent in the CG’s jump height, but here without affecting power output. Muscular power output, as jumping requires, is strongly associated with mobility and functional ability [35], factors impaired in CIPN patients. We thus propose to focus also on power training to alleviate functional disabilities in CIPN patients [42] and to counteract the CIPN-induced acceleration of neuromuscular degeneration.

The fact that both groups showed improvements suggests that both interventions are potentially effective in addressing different aspects of CIPN. However, the reader should take note that a placebo effect cannot be definitively ruled out in this study. As other RCTs have also demonstrated positive effects in their intervention groups by including an inactive control group [e.g. 32,34], we assume that the improvements we observed are genuine effects rather than placebo effects. Furthermore, we suppose that group differences in patients’ characteristic, i.e. age and amount of neurotoxic agents, may have influenced study results as discussed above. We therefore propose to stratify randomization according to those factors.

## Conclusions

We assume that endurance training contributed to a reduction in sensory symptoms in our study patients, while the balance part additionally affected the neuromuscular system relevant to patients’ functional status. This additional effect might reflect the IG’s superiority in the CIPN20 motor score, as well as in NtxS. However, we suspect that a larger sample is needed to reveal stronger group differences. Furthermore, we propose to integrate a third study arm with no physical intervention, and to expand upon CIPN diagnostics. We conclude that both exercises present a clear and relevant benefit for patients with CIPN by improving their functional status and alleviating CIPN symptoms. As pharmacological treatment options are very limited, these exercise interventions can be considered an effective non-pharmacological treatment approach. We are convinced that neuromuscular adaptation is possible despite CIPN, and that it’s never too late to start exercising.

## Abbreviations

CG: Control group
CIPN: Chemotherapy-induced peripheral neuropathy
CIPN20: Module of EORTC quality of life questionnaire
CPET: Cardiopulmonary exercise test
IAT: Individual anaerobic threshold
IG: Intervention group
MS_EO_: Monopedal stance
MS_EOunstable_: Monopedal stance on an unstable surface
NtxS: Neurotoxicity subscale of FACT&GOG
P_max_CPET_: Maximum power output during cardiopulmonary exercise test
P_max_jump_: Maximum power output during take-off
QoL: Quality of life
ST_EC_: Semi-tandem stance with eyes closed
ST_EO_: Semi-tandem stance with eyes open
W: Watt
